# Supplementary figures and images for: Botulinum toxin intoxication requires retrograde transport and membrane translocation at the ER in RenVM neurons
Source: eLife. 2024 Aug 28;12:RP92806. doi: 10.7554/eLife.92806 (PMC11357346; doi:10.7554/eLife.92806)

**Figure 1B**

**a-tRFP**

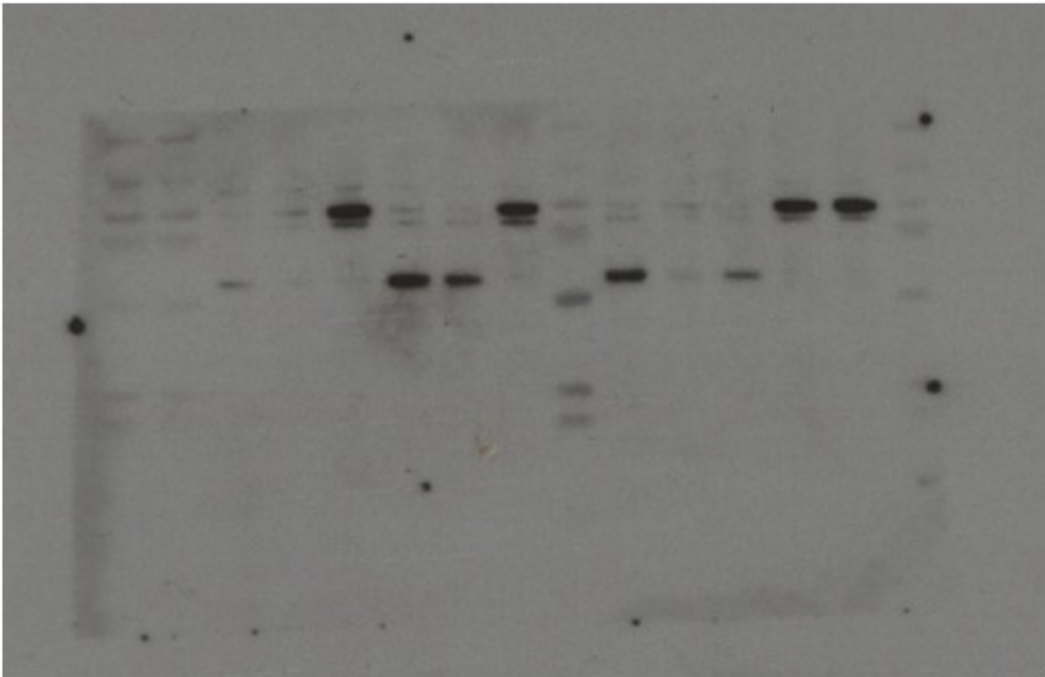

**a-tGFP**

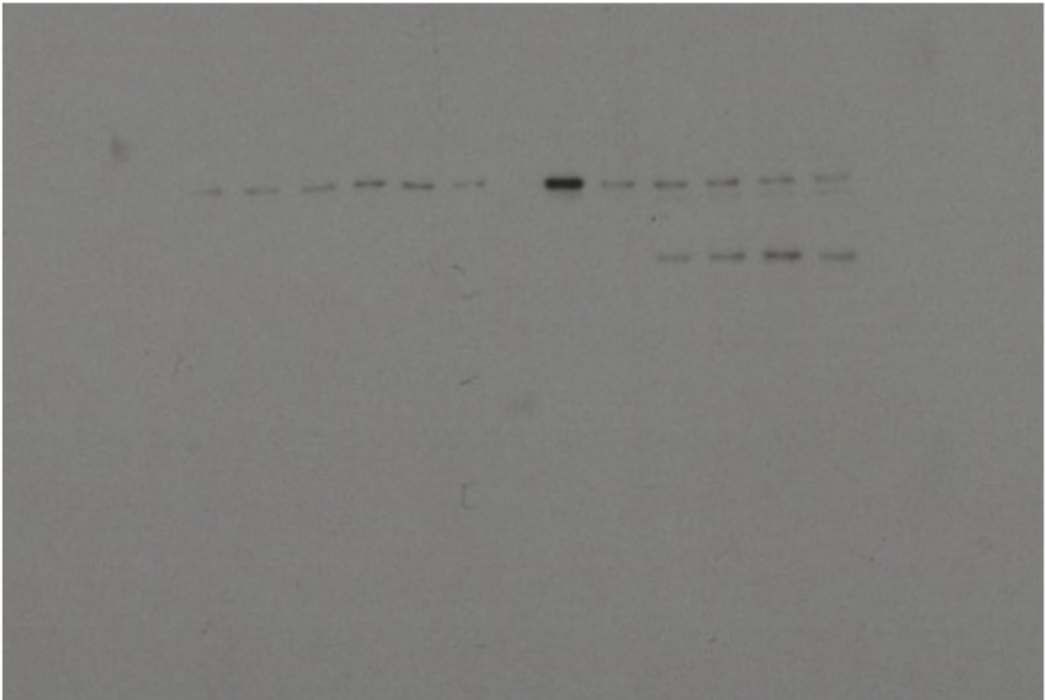

Supplement: Figure 1—source data 1. [file elife-92806-fig1-data1.pdf]

Figure 1B

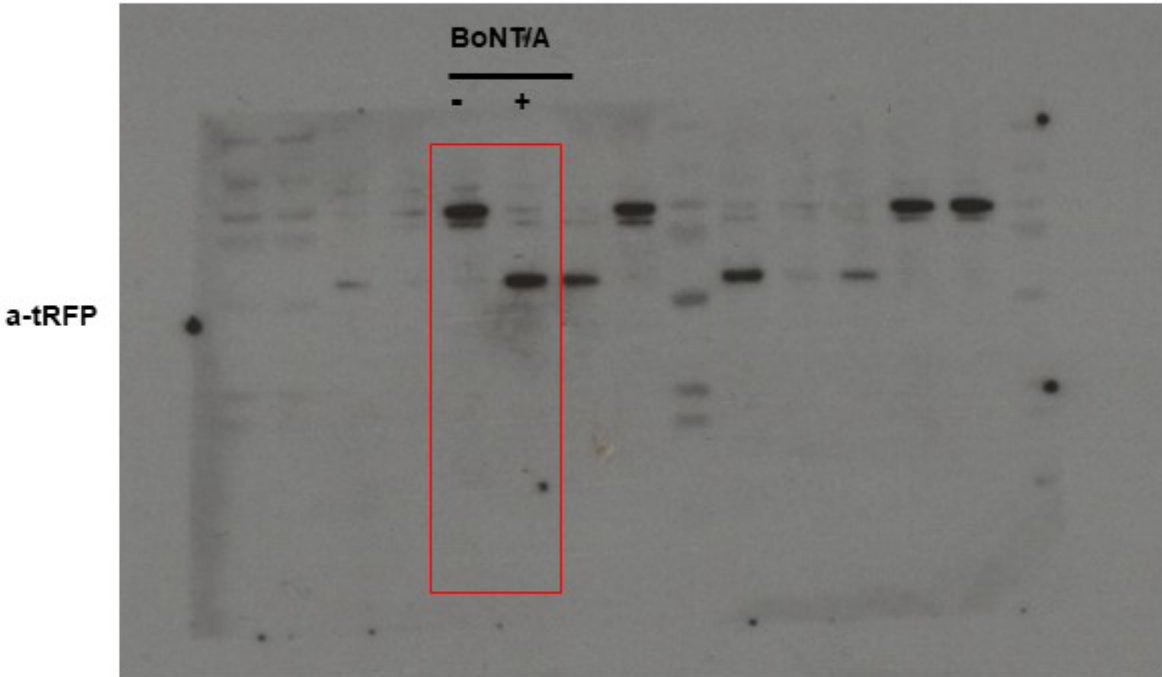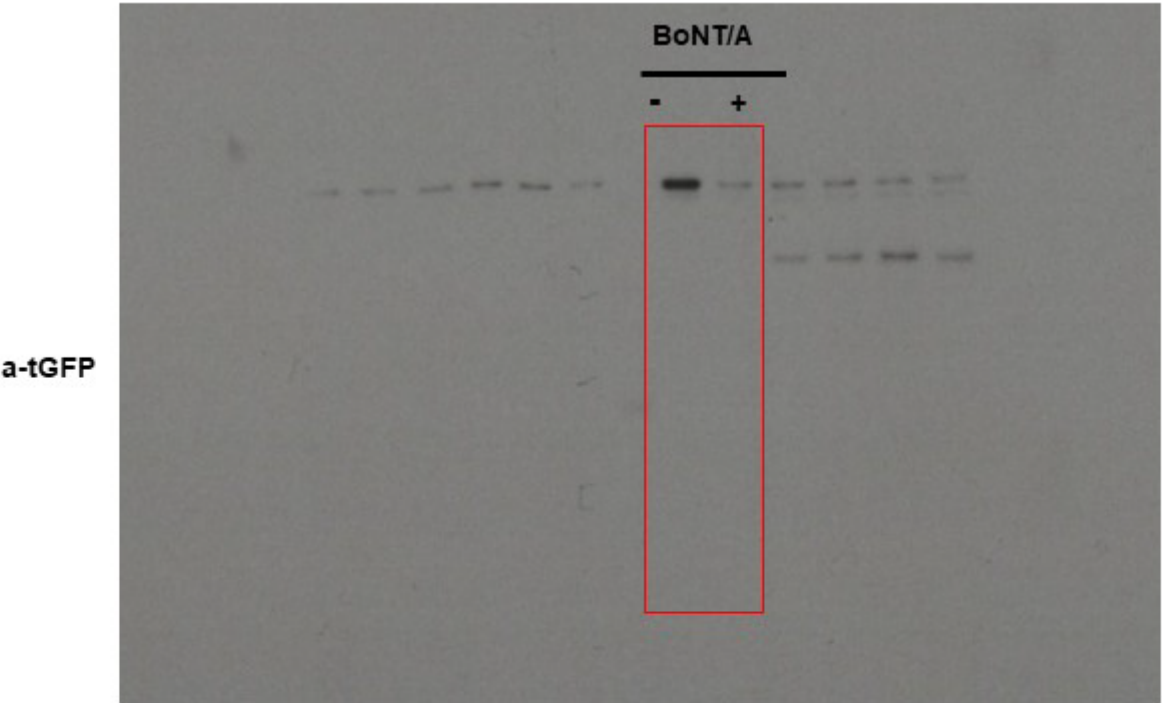

Supplement: Figure 1—source data 2. [file elife-92806-fig1-data2.pdf]

**Figure 1F**

**a-tRFP**

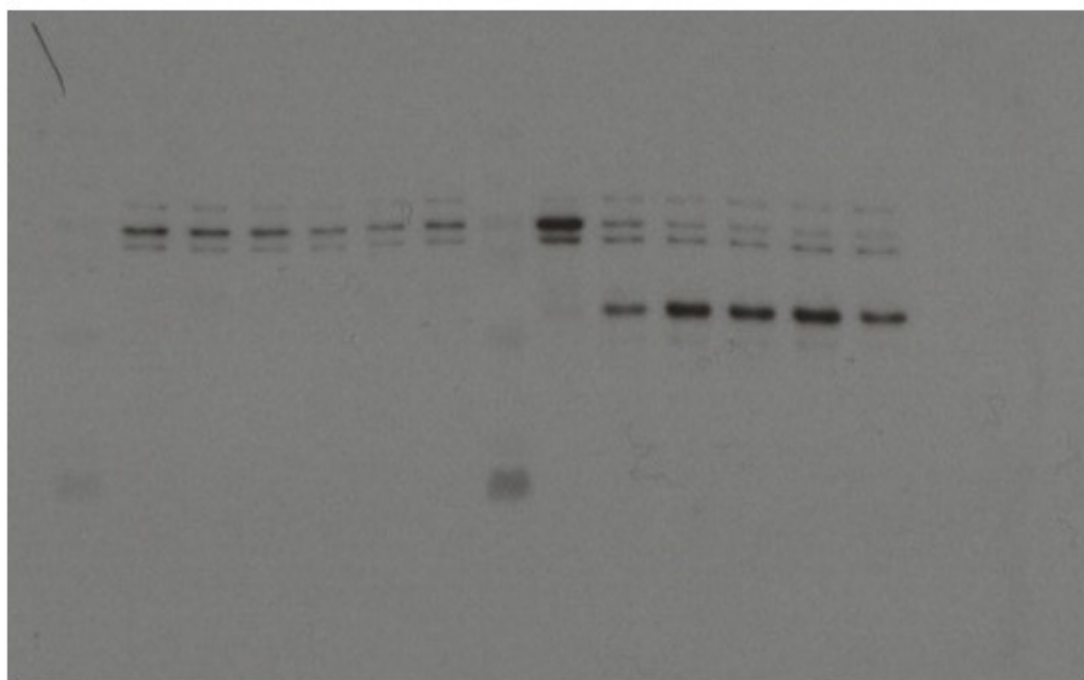

**a-tGFP**

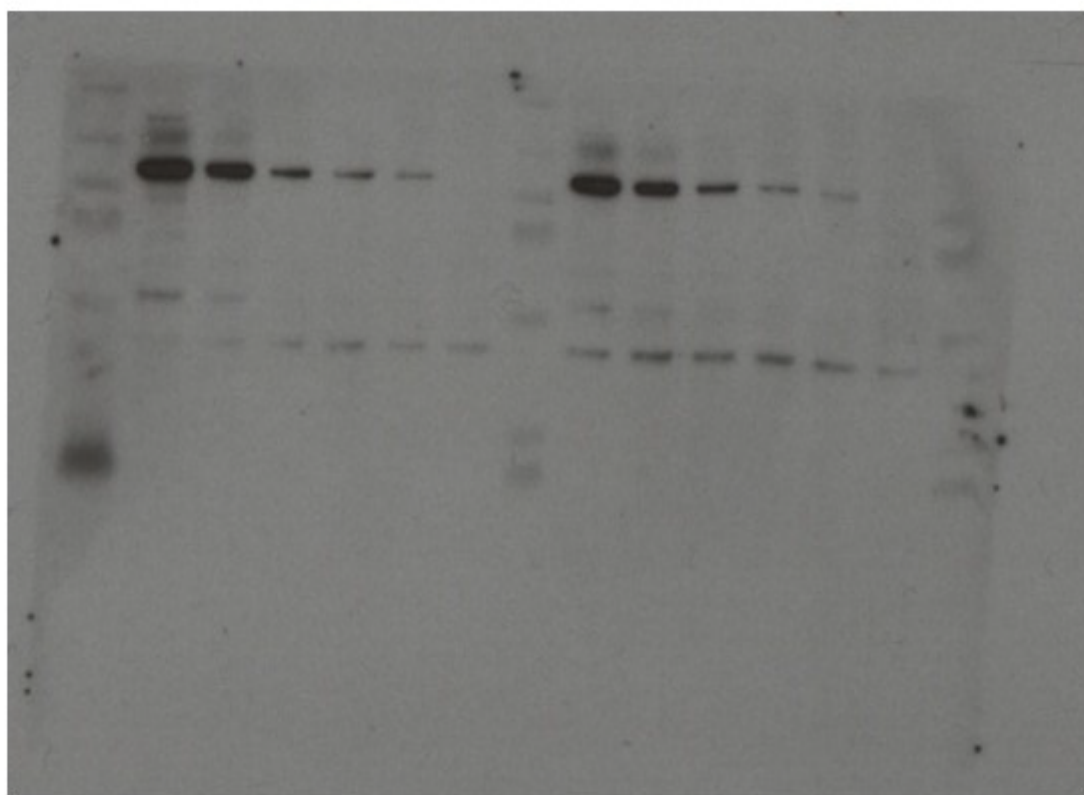

Supplement: Figure 1—source data 3. [file elife-92806-fig1-data3.pdf]

Figure 1F

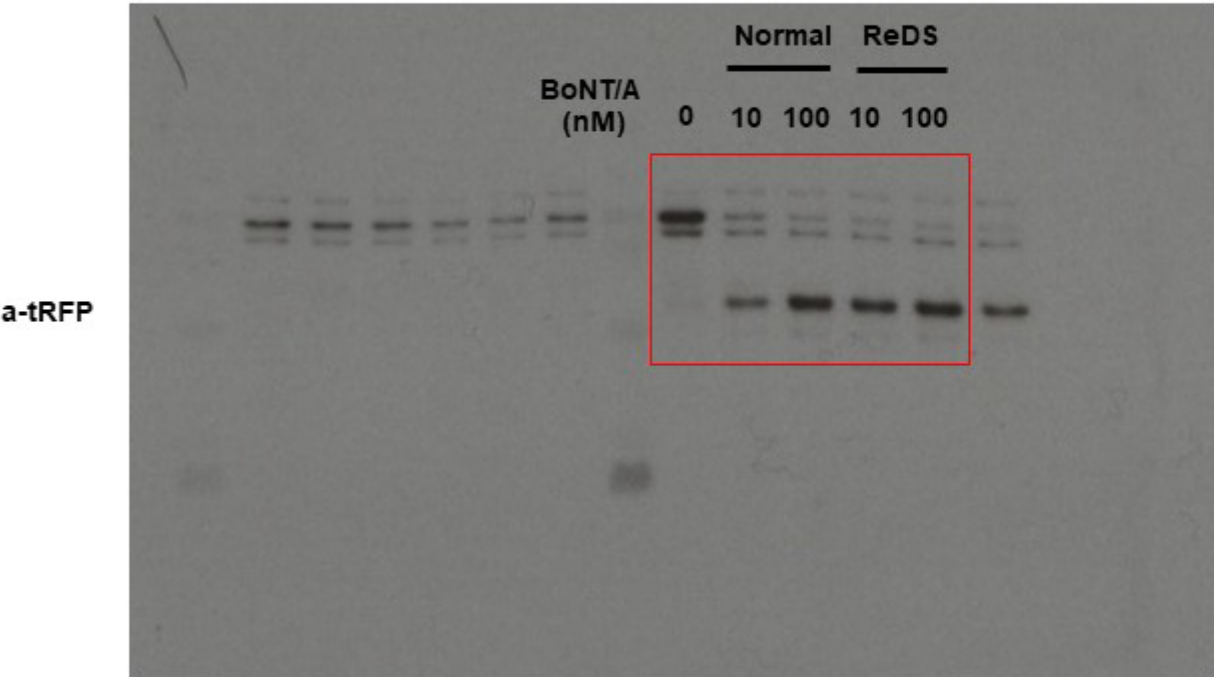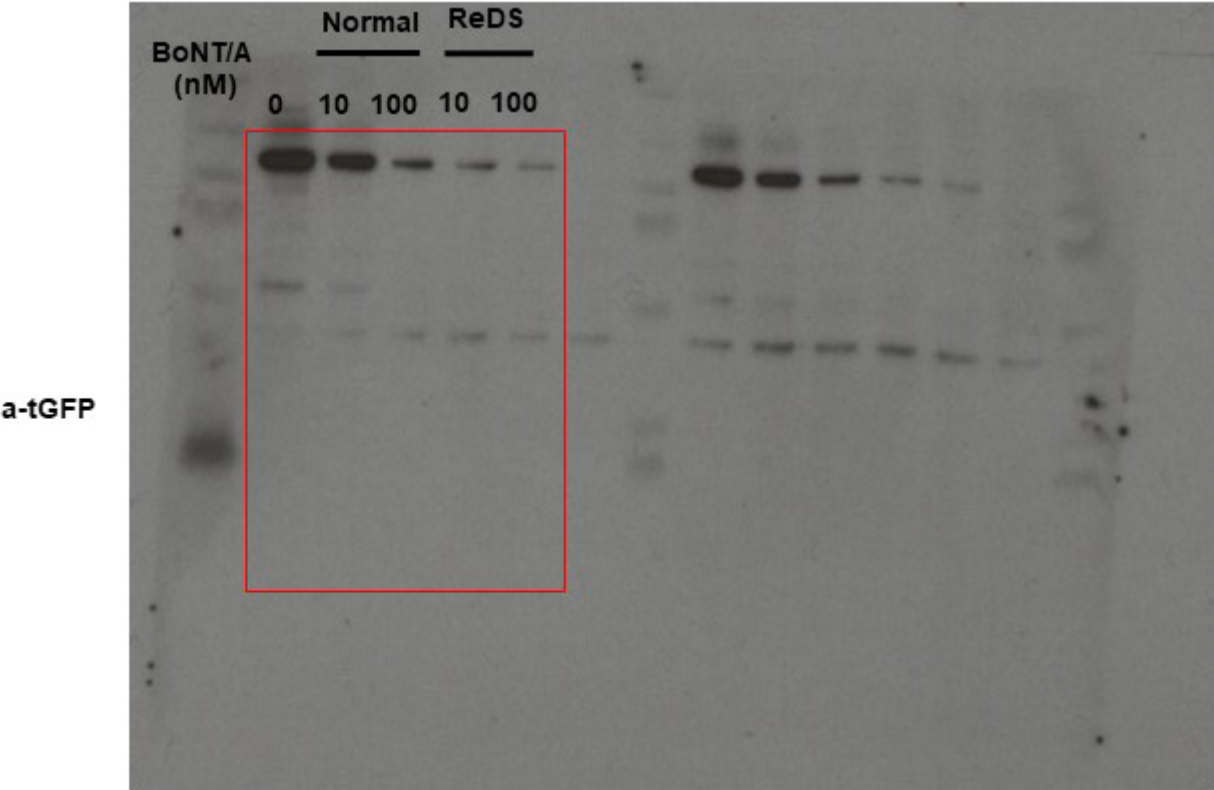

Supplement: Figure 1—source data 4. [file elife-92806-fig1-data4.pdf]
